# Mediating role of the body mass index in the prospective association between a healthy diet and evolution of asthma symptoms in elderly women

**DOI:** 10.1016/j.jarlif.2025.100011

**Published:** 2025-04-07

**Authors:** Wassila Ait-hadad, Annabelle Bédard, Laurent Orsi, Sébastien Chanoine, Orianne Dumas, Nasser Laouali, Nicole Le Moual, Bénédicte Leynaert, Valérie Siroux, Marie-Christine Boutron-Ruault, Raphaëlle Varraso

**Affiliations:** a Université Paris-Saclay, UVSQ, Univ. Paris-Sud, Inserm, Équipe d'Épidémiologie respiratoire intégrative, CESP Villejuif France; b Inserm U1209, CNRS, University Grenoble Alpes, Institute for Advanced Biosciences (IAB), Team of Environmental Epidemiology applied to the Development and Respiratory Health Grenoble France; c Université Paris-Saclay, UVSQ, Univ. Paris-Sud, Inserm, Gustave Roussy, Équipe "Exposome et Hérédité", CESP, 94805, Villejuif, 94800, France; d Institute of Biological Sciences (ISSB), UM6P Faculty of Medical Sciences, Mohammed VI Polytechnic University, Ben Guerir, Morocco Department of Biostatistics and Epidemiology, Morocco; e School of Public Health and Health Sciences, University of Massachusetts, Amherst, MA, USA

**Keywords:** AHEI-2010, Asthma symptoms incidence, Change in asthma symptom, Body mass index, Mediation analyses, Counterfactual framework

## Abstract

**Objectives:**

Diet and obesity exhibit complex interrelationships with asthma, particularly among elderly women. We aimed to clarify the impact of healthy diet assessed by the Alternate Healthy Eating Index-2010 (AHEI-2010) on: 1) the incidence of asthma symptoms, and 2) among women with symptoms in 2011, the change in asthma symptoms, while accounting for the potential mediating role of BMI.

**Design:**

A nested case-control study on asthma with follow-up data.

**Setting:**

Within the French E3N cohort.

**Participants:**

8621 elderly women (62 years on average in 1993)

**Measurements:**

Dietary data were collected in 1993 and 2005 using semi-quantitative questionnaires. Using the validated asthma symptom score assessed in 2011 and 2018, asthma symptom incidence among women with no asthma symptom in 2011 (*n* = 5700) and change in asthma symptoms (reduced, stable, increased) among those with asthma symptoms in 2011 (*n* = 2921) were defined. BMI was calculated in 2008. Marginal structural models were used to estimate total, direct and indirect effects mediated by BMI.

**Results:**

After adjustment for potential confounders, we found a significant indirect effect of healthier diet on lower risk of asthma symptoms incidence mediated by lower BMI (OR for AHEI-2010 quintile 5 vs quintile 1 = 0.95 (0.92–0.97)), without significant total (OR=0.87 (0.66–1.10)) nor direct (OR=0.92 (0.71–1.15)) effects. Among women with asthma symptoms, we also found a significant indirect effect of healthier diet on reduced asthma symptoms mediated by lower BMI (OR for AHEI-2010 >median *vs* ≤median=1.02 (1.00–1.03)) without significant total (OR=1.12 (0.94–1.34)) nor direct effects (OR=1.10 (0.93–1.31)).

**Conclusion:**

A healthy diet was associated with reduced risk of asthma symptoms over time, partly through a lower BMI.

## 1 Introduction

Asthma, a chronic inflammatory disease, is common in the elderly and is of special concern in older women, who tend to have more severe asthma than older men [1]. Several epidemiological studies have reported that a healthy diet, associated with lower inflammatory biomarkers [2] and more recently with an increase in short-chain fatty acids [3] known to reduce airway inflammation [4], could improve asthma outcomes among children and adults [5], but its impact among elderly women has been little studied [6,7]. In our previous work, based on cross-sectional data, we reported that healthy diet, estimated by the Alternate Healthy Eating Index-2010 (AHEI-2010) was associated with lower asthma symptoms score, whereas higher body mass index (BMI) was associated with greater asthma symptoms score [6]. However, longitudinal data are needed to properly disentangle the effect of healthier diet on asthma unexplained by obesity from the effect explained by obesity. Indeed, as obesity is a likely risk factor for asthma, and that better diet quality has been associated with a lower risk of obesity, it is reasonable to posit that BMI could act as a mediator rather than a confounder in this association [5]. Using mediation analyses in the counterfactual framework and longitudinal data, we previously reported that a healthy Plant-Based Diet (hPDI) was associated with a reduced risk of asthma incidence over time, partly mediated by BMI [7]. Another diet index that emphasises healthy diet through plant-based foods is the AHEI-2010, which provides higher scores for healthy plant-based foods but also for some animal-sourced foods (such as fish) and was associated with lower levels of inflammatory biomarkers [2] and with a lower risk of major chronic disease [8]. Therefore, we aimed to clarify the impact of the AHEI-2010 among elderly women on 1) the incidence of asthma symptoms, and 2) among women with asthma symptoms, the change in asthma symptoms, while accounting for the potential mediating role of BMI.

## 2 Methods

Asthma-E3N, a nested case–control study on asthma within the E3N cohort (*n* = 98,995 women included in 1990, follow-up every 2–3 years [9]), was conducted in 2011 (*n* = 19,404 women, 91 % response rate) with follow-up data in 2018 (*n* = 15,301). We used the mean of the AHEI-2010 [8] from two validated semi-quantitative food history questionnaires in 1993 and 2005, and categorised into quintiles or according to the median value. We excluded women with no asthma symptom data in 2011 or 2018 (*n* = 5004), those without dietary data in 1993 or 2005 (*n* = 1676), and women reporting asthma symptoms in 2011 (*n* = 2921), resulting in a final sample of 5700 women. All methodological details are available in our previous publications [6,7].

The asthma symptoms score, that captures the heterogeneity and variability of the disease and is based on the number of positive answers to 5 respiratory symptoms occurring during the past 12 months, was validated as continuous measure of asthma in epidemiological studies [10] and evaluated in 2011 and 2018. BMI, based on self-reported current weight and height and expressed in kg/m², was calculated in 2008 with the “last observation carried forward” method to take care of missing data, and analysed as a continuous variable. Analyses were performed among women from Asthma-E3N who answered questions on the asthma symptom score in 2011 and 2018 (*n* = 10,297) and with dietary data in 1993 and in 2005 (*n* = 8621).

For aim 1, women with no asthma symptom in 2011 and 2018 served as the reference group (*n* = 5149), and women with no symptom in 2011 and at least one asthma symptom in 2018 as “incident” (*n* = 551). For aim 2, the change in asthma symptoms was evaluated among symptomatic women in 2011, as “stable” if the difference between the 2018 and the 2011 scores was null (reference, *n* = 730), “increased” if the difference was positive (*n* = 241) and “reduced” if it was negative (*n* = 1950).

**Fig. 1:**
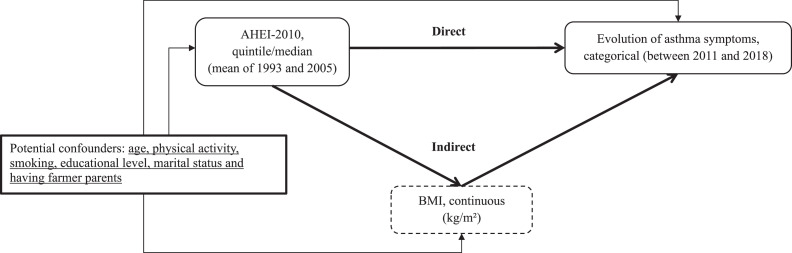
Direct effect of healthy diet on evolution of asthma symptoms and indirect effect mediated by BMI.

To disentangle the direct effect of a healthy diet on evolution of asthma symptoms (OR_DE_) from the indirect effect mediated by BMI (OR_IE_), we applied marginal structural models (MSMs), as proposed by Lange et al. [11], in particular because it can be used for all types of variables, adjusted for age, smoking, physical activity, education, marital status, and having farmer parents (Fig. 1). As shown in Fig. 1, we ensured that the temporal sequence between exposure, mediator, and outcome was respected.

To account for potential residual confounding by smoking (for example smoking intensity and/or duration), we conducted stratified analyses according to smoking status (never-smokers and ever-smokers), and tested the statistical significance of interaction term between AHEI-2010 and smoking status. We also conducted several sensitivity analyses consisting in: (1) models further adjusted for total energy intake, which was not included in the main analyses to avoid an isocaloric substitution among foods [12]; (2) models using BMI collected in 2005, instead of using BMI collected in 2008; (3) Given the high prevalence of asthma-related multimorbidity, we excluded women with cancers or cardiovascular diseases (CVD) in 1993; 4) models further adjusted for the asthma symptoms score in 2011 for aim 2. All analyses were performed using SAS version 9.4 (SAS Institute Inc.).

## 3 Results

Women were aged 62 years on average in 1993. Among women with no asthma symptom in 2011 (*n* = 5700), 9.7 % reported at least one asthma symptoms in 2018; and among women with at least one asthma symptoms in 2011 (*n* = 2921), 8 % reported more symptoms in 2018 (increased), 25 % were stable and 67 % reported fewer symptoms (reduced). After adjustment for age, women in the highest quintile of the AHEI-2010 consumed less energy, were more physically active, were less likely to be current smokers, had more often farmer parents and were less often overweight/obese (BMI≥25), as compared to women in the lowest quintile of the AHEI-2010.

**Fig. 2:**
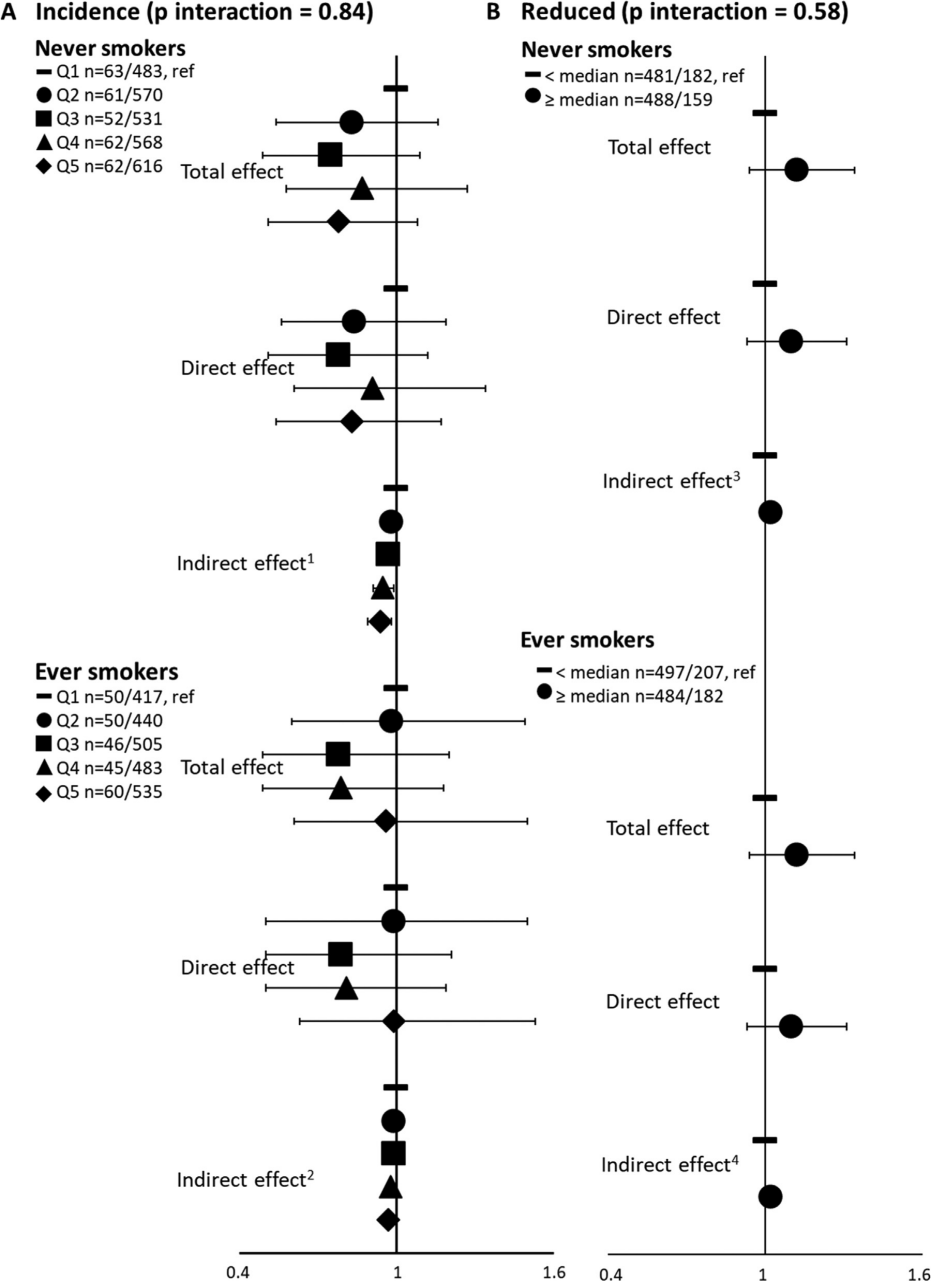
Associations between categories (quintiles or binary) of the AHEI- score with the incidence of asthma symptoms and the change in asthma symptoms among symptomatic women, according to smoking status. Ref = reference. Models were adjusted for age, physical activity, educational level, marital status, and having farmer parents. ^1^OR (95CI) of indirect effect for incidence symptoms among never-smokers: 0.94 (0.89–0.98) ^2^OR (95CI) of indirect effect for incidence symptoms among ever-smokers: 0.97 (0.94–0.99) ^3^OR (95CI) of indirect effect for reduced symptoms among never-smokers: 1.02 (0.99–1.04) ^4^OR (95CI) of indirect effect for reduced symptoms among ever-smokers: 1.02 (1.00–1.04).

After adjustment for confounders, we observed a significant indirect effect between healthier diet and decreased incidence of asthma symptoms mediated by a lower BMI, with a significant dose-response relationship (Table 1); the proportion mediated by BMI of the association between healthier diet and incidence of asthma symptoms accounted for 35 % of the total effect. The direct and the total effects of healthier diet on incident asthma symptoms were not statistically significant, although the ORs were lower than one. Sensitivity analyses led to similar results when: 1) adjusting for total energy intake (OR_IE_ (95 %CI) for quintile 5 (Q5) *vs* quintile 1 (Q1)=0.96 (0.94–0.98)); 2) using BMI in 2005 (OR_IE_ for Q5 vs Q1=0.95 (0.92–0.97)); 3) excluding women with cancers or CVD (OR_IE_ for Q5 vs Q1=0.97 (0.95–0.99)), with proportions mediated by BMI varying between 17 and 25 %. Analyses stratified by smoking status showed similar results with a significant inverse indirect effect mediated by BMI both in never-smokers (OR_IE_ for Q5 vs Q1=0.94 (0.89–0.98)) and ever-smokers (OR_IE_ for Q5 vs Q1=0.97 (0.94–0.99)) (p for interaction=0.84) (Fig. 2A).

**Table 1:** Associations between the AHEI-2010 score with the incidence of asthma symptoms and the change of asthma symptoms among symptomatic women, mediated by BMI.

AHEI-2010	No.	Total effect	Direct effect	Indirect effect	Proportion mediated
	**Among women with no asthma symptom in 2011**				
	Incident *vs.* no symptom*				
		OR (95 %CI)	OR (95 %CI)	OR (95 %CI)	

Continuous	551/5149	0.93 (0.82–1.05)	0.95 (0.84–1.06)	0.98 (0.94–1.00)	35 %
Quintile 1	113/900	1.00 (ref)	1 .00 (ref)	1 .00 (ref)	
Quintile 2	111/1011	0.99 (0.77–1.25)	1.00 (0.78–1.26)	***0.99 (0.98–0.99)***	
Quintile 3	98/1036	0.89 (0.68–1.13)	0.92 (0.70–1.16)	***0.97 (0.96–0.98)***	
Quintile 4	107/1051	0.83 (0.63–1.05)	0.87 (0.66–1.10)	***0.96 (0.94–0.98)***	
Quintile 5	122/1151	0.87 (0.66–1.10)	0.92 (0.71–1.15)	***0.95 (0.92–0.97)***	

	**Among symptomatic women (asthma symptom score ≥1)**#				
	**Increased *vs.* Stable**				
Continuous	241/730	1.15 (0.95–1.36)	1.14 (0.95–1.34)	1.01 (0.95–1.06)	7 %
≤ median	127/389	1 .00 (ref)	1 .00 (ref)	1 .00 (ref)	
> median	114/341	1.04 (0.78–1.39)	1.02 (0.76–1.36)	1.01 (0.99–1.04)	

	**Reduced *vs.* Stable**				
Continuous	1950/730	1.05 (0.94–1.18)	1.04 (0.94–1.15)	1.01 (0.98–1.05)	20 %
≤ median	978/389	1 .00 (ref)	1 .00 (ref)	1 .00 (ref)	
> median	972/341	1.12 (0.94–1.34)	1.10 (0.93–1.31)	***1.02 (1.00–1.03)***	

Ref = reference.

Odds ratio (OR) and 95 % confidence interval (CI) were estimated from marginal structural models for an increase of 1 category (quintile or binary) in the AHEI-2010; 95 % CI were obtained from 500 bootstrapped samples.

The total effect represents the overall effect of the exposure (diet) on the disease (asthma); the indirect effect represents the effect passing through the mediator (body mass index); and the direct effect represents the effect unexplained by the mediator.

Models were adjusted for age, physical activity, smoking, educational level, marital status and having farmer parents.

⁎ Women with no symptom (reference): *n* = 5149; mean age=62 years; 46 % ever-smokers; 24 % overweight or obese (BMI≥25 kg/m^2^). Incident: *n* = 551; mean age=63 years; 46 % ever-smokers; 32 % overweight or obese (BMI≥25 kg/m^2^).

# Stable (reference): *n* = 730; mean age=63 years; 53 % ever-smokers, 42 % overweight or obese (BMI≥25 kg/m^2^). Increased: *n* = 241; mean age=63 years; 51 % ever-smokers; 39 % overweight or obese (BMI≥25 kg/m^2^). Reduced: *n* = 1 950; mean age=62 years; 50 % ever-smokers; 39 % overweight or obese (BMI≥25 kg/m^2^).

Among women with asthma symptoms in 2011 (*n* = 2921), we found a positive significant indirect effect mediated by BMI between healthier diet and reduced asthma symptoms, accounting for 20 % of the total effect (Table 1). The direct and total effects of healthier diet on reduced asthma symptoms were not statistically significant, although the ORs were greater than 1. Sensitivity analyses led to similar results when: 1) adjusting for total energy intake (OR_IE_ for >median *vs.* ≤median=1.01 (1.00–1.03)); 2) using BMI in 2005 (OR_IE_ for >median *vs*. ≤median=1.02 (1.00–1.03)); 3) excluding women with cancers or CVD (OR_IE_ for >median *vs*. ≤median=1.01 (1.00–1.03)); and 4) adjusting for the asthma symptoms score in 2011 (OR_IE_ for >median *vs*. ≤median=1.02 (1.00–1.03)). After stratification on smoking (Fig. 2B), the positive indirect effect was significant only in ever-smokers (OR_IE_ for >median *vs* ≤median=1.02 (0.99–1.04), and 1.02 (1.00–1.04) in never- and ever-smokers, respectively), although the interaction was not significant (p for interaction=0.58). By contrast, no association was observed between the AHEI-2010 score and increased asthma symptoms.

## 4 Discussion

We found that the effect of healthier diet both on the decreased risk of asthma symptoms incidence and on the reduced number of symptoms over 7 years is partly mediated by a lower BMI. Although total and direct effects were not statistically significant, we reported that indirect paths by BMI are likely a more accurate way to explain the association between healthier diet and asthma among elderly women. These findings suggest that BMI plays a key mediating role in the diet-asthma relationship (35 %), while other mechanisms could also contribute. Indeed, the AHEI-2010 reflects overall dietary quality, incorporating food groups with anti-inflammatory and antioxidant properties (*e.g.*, fruits, vegetables, whole grains, and fish) that may influence asthma outcomes, and components such as polyphenols, omega-3 fatty acids, and fiber have been linked to reduced airway inflammation and improved lung function [13]. Although we did not incorporate inflammatory biomarkers, it has been extensively reported that a higher consumption of plant-based food like fruits, vegetables, legumes and whole grains, evaluated especially by the AHEI-2010, is associated with lower inflammatory biomarkers such as CRP, IL-6 et TNF-α [2] and more recently with an increase in short-chain fatty acids (produced by bacteria in the gut during fermentation of fibre from dietary plant matter) [3] known to reduce airway inflammation [4].

Regarding the incidence of symptoms, the indirect effect mediated through BMI accounted for 35 % of the total effect, which is consistent with our previous work where we found a percentage of the association between the incidence of asthma symptoms and the hPDI mediated by BMI of 35 % [7]. Using several diet scores helps to capture the variability and diversity of different diet types. Indeed, the AHEI-2010 and the hPDI both reflect healthy diet, even if the correlation between AHEI-2010 and hPDI was “only” 60 % suggesting that both scores bring complementary information.

Regarding reduced asthma symptoms, the indirect effect mediated through BMI accounted only for 20 % of the total effect. Only one previous study found that healthier diet was directly associated with reduced asthma symptoms over time in never-smokers only, with a non-significant indirect effect through BMI accounting for 5 % of the total effect [14]. Differences in the study populations (middle-aged men and women in the previous study *vs* older women in ours), as well as differences in the outcome classification for the stable group (the previous study categorized participants without asthma symptoms in the stable group, whereas we defined the stable group only among women with asthma symptoms at baseline) may explain discrepancies between studies.

Our study has several strengths and limitations. Our study was longitudinal with a large sample size, which allows accounting for several potential confounders, and performing stratified analyses to address the robustness of the findings, although subsample sizes were limited in some categories. Diet was evaluated in 1993 and 2005, and asthma symptoms score in 2011 and 2018. Although participants may have modified their diet between 1993 and 2011, we used the average of diet score in 1993 and 2005. We ensured the correct temporal sequence: diet was assessed before BMI, and BMI before asthma symptoms. However, some limitations must be acknowledged: (i) dietary changes between 1993 and 2011 may not have been fully captured, though diet was assessed using validated food frequency questionnaires [15]; (ii) asthma symptoms were assessed at only two time points, but we relied on a validated asthma symptoms score [10]; (iii) BMI may have changed over time, yet similar findings were observed using BMI from 2005, which was highly correlated with BMI in 2008 (*r* = 0.91) and showed stable obesity prevalence (5.3 % *vs.* 6.0 %). Although MSMs, are robust to time-dependent confounding, they rely on strong assumptions, including the absence of unmeasured confounders for the exposure-outcome, exposure-mediator, and mediator-outcome relationships, as well as the absence of variables that are both effects of the exposure and confounders of the mediator-outcome relationship [16]. It is also likely that other factors, such as obesity-related multimorbidities may have affected asthma outcomes, but we found similar results when we excluded women with cardiovascular and cancer comorbidities. Total energy intake may lie on the causal pathway between diet and asthma [12] and to avoid any isocaloric substitution of foods, our main models were not adjusted for total energy. However, we performed sensitivity analyses adjusted on total energy intake, with results similar to the main model. Among women with symptoms in 2011, to take into account the difference in starting point between the participants, we further adjusted for the asthma symptoms score in 2011, and the results were similar. As the AHEI-2010 diet score is associated with chronic obstructive pulmonary disease (COPD) [17], the potential overlap between asthma and COPD could contribute to the association between AHEI-2010 and asthma especially in this aged population, but associations were not modified by smoking status. Moreover, the asthma symptoms score is particularly appropriate for longitudinal studies, capturing both asthma incidence and disease variability in women with asthma and it relates to symptoms specific to asthma and not of COPD. Finally, the relative homogeneity of the studied population actually helps with causal inferences by reducing residual potential confounding.

A healthy diet was associated with a lower risk of asthma incidence and a reduced number of asthma symptoms over time partly mediated by a lower BMI highlighting the need to promote obesity prevention and healthy dietary choices through multi-intervention programs, as advocated by several national dietary guidelines [18], to support asthma prevention in elderly women. And beyond body composition, future studies should investigate alternative mechanisms by integrating biomarkers of systemic inflammation and gut microbiota composition to better understand the mechanisms linking diet and asthma, while also using MSMs to assess multiple mediators simultaneously and provide a more comprehensive analysis of this relationship.

## Funding

This work was supported by the Institut pour la Recherche en Santé Publique (IReSP/JPM/MG-2013-0189), and of the joint help of Direction Générale de la Santé (DGS), the Mission recherche de la Direction de la Recherche, des Etudes, de l'Evaluation et des Statistiques (Mire-DREES), the Caisse nationale d'assurance maladie des travailleurs salariés (CNAMTS), Régime Social des Indépendants (RSI) & Caisse nationale de solidarité pour l'autonomie (CNSA). The E3N-E4N cohort is supported by the Mutuelle Générale de l'Education Nationale (MGEN); the French League against Cancer (LNCC); Gustave Roussy; and the French Research Agency (ANR grant, ANR-10-COHO-0006). WHA was supported by a doctoral fellowship from the Ecole Doctorale de Santé Publique, Paris-Saclay University, France.

## Ethics approval

The E3N study was authorized by the French National Commission for Data Protection and Privacy (CNIL n°106.246). E3N data enrichment with the MGEN database was granted ethical approval (CCTIRS n°13.794) and was authorized by the CNIL (n°327346V14).

## CRediT authorship contribution statement

**Wassila Ait-hadad:** Writing – original draft, Validation, Software, Methodology, Formal analysis, Conceptualization. **Annabelle Bédard:** Validation. **Laurent Orsi:** Writing – review & editing, Methodology. **Sébastien Chanoine:** Writing – review & editing. **Orianne Dumas:** Writing – review & editing. **Nasser Laouali:** Writing – review & editing. **Nicole Le Moual:** Writing – review & editing. **Bénédicte Leynaert:** Writing – review & editing. **Valérie Siroux:** Writing – review & editing. **Marie-Christine Boutron-Ruault:** Writing – review & editing, Writing – original draft, Visualization, Validation, Supervision, Resources, Investigation, Conceptualization. **Raphaëlle Varraso:** Writing – review & editing, Writing – original draft, Visualization, Validation, Supervision, Software, Methodology, Funding acquisition, Formal analysis, Conceptualization.

## Declaration of competing interest

The authors declare the following financial interests/personal relationships which may be considered as potential competing interests:

Sebastien Chanoine reports a relationship with Boehringer Ingelheim France SAS that includes: non-financial support. If there are other authors, they declare that they have no known competing financial interests or personal relationships that could have appeared to influence the work reported in this paper.
